# Distribution of ticks in the Western Palearctic: an updated systematic review (2015–2021)

**DOI:** 10.1186/s13071-023-05773-6

**Published:** 2023-04-24

**Authors:** Madeleine Noll, Richard Wall, Benjamin L. Makepeace, Hannah Rose Vineer

**Affiliations:** 1 Institute of Veterinary and Ecological Sciences, University of Liverpool, Liverpool, UK; 2 School of Biological Sciences, University of Bristol, Bristol, UK

**Keywords:** Ticks, Systematic review, Spatial analysis, Western Palearctic

## Abstract

**Background:**

The distributions of ticks and tick-borne pathogens are thought to have changed rapidly over the last two decades, with their ranges expanding into new regions. This expansion has been driven by a range of environmental and socio-economic factors, including climate change. Spatial modelling is being increasingly used to track the current and future distributions of ticks and tick-borne pathogens and to assess the associated disease risk. However, such analysis is dependent on high-resolution occurrence data for each species. To facilitate such analysis, in this review we have compiled georeferenced tick locations in the Western Palearctic, with a resolution accuracy under 10 km, that were reported between 2015 and 2021

**Methods:**

The PubMed and Web of Science databases were searched for peer-reviewed papers documenting the distribution of ticks that were published between 2015 and 2021, using the Preferred Reporting Items for Systematic Reviews and Meta-Analyses (PRISMA) guidelines. The papers were then screened and excluded in accordance with the PRISMA flow chart. Coordinate-referenced tick locations along with information on identification and collection methods were extracted from each eligible publication. Spatial analysis was conducted using R software (version 4.1.2).

**Results:**

From the 1491 papers identified during the initial search, 124 met the inclusion criteria, and from these, 2267 coordinate-referenced tick records from 33 tick species were included in the final dataset. Over 30% of articles did not record the tick location adequately to meet inclusion criteria, only providing a location name or general location. Among the tick records, *Ixodes ricinus* had the highest representation (55%), followed by *Dermacentor reticulatus* (22.1%) and *Ixodes frontalis* (4.8%). The majority of ticks were collected from vegetation, with only 19.1% collected from hosts.

**Conclusions:**

The data presented provides a collection of recent high-resolution, coordinate-referenced tick locations for use in spatial analyses, which in turn can be used in combination with previously collated datasets to analyse the changes in tick distribution and research in the Western Palearctic. In the future it is recommended that, where data privacy rules allow, high-resolution methods are routinely used by researchers to geolocate tick samples and ensure their work can be used to its full potential.

**Graphical Abstract:**

**Figure:**
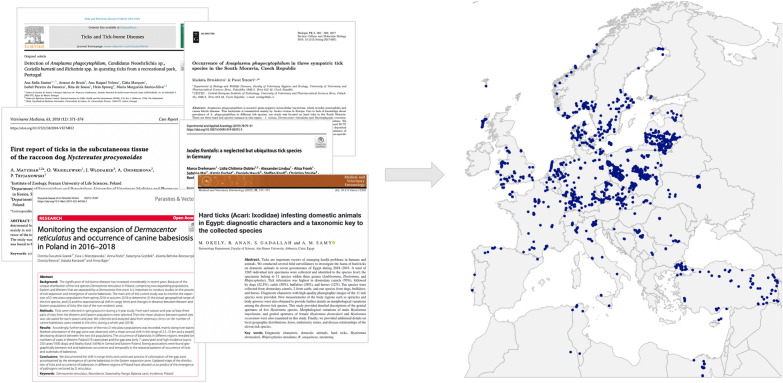

**Supplementary Information:**

The online version contains supplementary material available at 10.1186/s13071-023-05773-6.

## Background

Ticks are obligate hematophagous arthropods of global importance due to their public and veterinary health impacts; their blood-feeding can cause irritation, secondary infection, allergic reactions and, in some cases, paralysis [1]. However, their ability to transmit a wide range of pathogens, including viruses, protozoa and bacteria, makes them of particular importance [2]. For these reasons it is imperative to understand the distribution of individual tick species across the Western Palearctic, to guide future research as well as risk assessment and mitigation.

A total of 66 tick species, all belonging to the Ixodidae and Argasidae families, are endemic in the Western Palaearctic (11°W to 45°E and 29°S to 71°N). These ticks belong to five genera of Ixodidae, namely *Ixodes* (28 species), *Hyalomma* (9), *Rhipicephalus* (8), *Haemaphysalis* (7) and* Dermacentor* (2), and to two genera of Argasidae, namely *Argas* (5) and *Ornithodoros* (7) [3]. Although all are found in the Western Palearctic, the distribution of individual species varies according to their climatic niche. For example, *Ixodes ricinus* is a generalist that is present across much of the Western Palearctic, ranging from North Africa to Scandinavia and from Ireland to Russia [4]. Conversely, *Hyalomma marginatum* has a more restricted distribution around the Mediterranean basin [5]. However, a range of environmental and socio-economic factors, including climate change, have resulted in recent changes in the distribution and epidemiology of many tick species and tick-borne diseases. Tick species showing such changes in distribution include *I. ricinus, *whose range has expanded northwards in Sweden since the 1980s, from approximately 61°N to 66°N [6–8], and *Dermacentor reticulatus*, whose range has expanded across central and north-eastern Europe [9, 10]. The incidence of tick-borne disease has mirrored the range expansion of their vectors [11]. Since these changes have direct veterinary and public health implications, there has been a growing interest in surveillance to determine the current distribution of these ticks, as well as mechanistic and correlative models to assess likely future changes [12–14].

Geostatistical and spatial analysis, such as species distribution modelling, requires accurate species occurrence data. Previous efforts have been made to compile localised tick occurrence data, but several of these datasets provide tick distributions delimited by political boundaries, such as those provided by the European Centre for Disease Prevention and Control, which are not adequate for some spatial analyses. Furthermore, despite increases in the number of schemes encouraging the reporting of ticks and their locations by the public, the identification of these specimens may not be reliable. As a result, we designed the present systematic literature review to combine the results of existing peer-reviewed primary publications investigating tick distributions between 1970 and 2014 [15, 16], with the aim to create a secondary dataset of localised geographical occurrences in the Western Palearctic between 2015 and 2021. Our overall purpose is to provide a freely available updated set of records for tick species in the Western Palearctic for researchers investigating their spatial distribution.

## Methods

### Search strategy

The Preferred Reporting Items for Systematic Reviews and Meta-Analyses (PRISMA) guidelines [17] were followed in designing and performing this systematic review. The relevant literature was largely found by searching the Web of Science [18] and PubMed [19] databases, although we included other eligible literature when identified. The search was carried out in English using the key word string employed by European Food Safety Authority Panel on Animal Health and Welfare [20] to identify titles and/or abstracts from papers published between 1 January 2015 and 31 December 2021. All references were imported into a Microsoft Excel (Microsoft Corp., Redmond, WA, USA) spreadsheet for assessment by the lead author.

### Criteria for inclusion and data extraction

Following the primary literature search of the databases and the identification of any additional relevant papers, all duplicates were removed. The literature was then initially screened for relevance based on the title and abstract, following which selected papers were downloaded and subject to a second screening to check their eligibility (see Table 1 for inclusion criteria). The following data were extracted from the eligible studies: (i) tick genus and species; (ii) identification method; (iii) country, named location and geographical longitude and latitude of found ticks (converted into degrees decimal if necessary); and (iv) the collection method and host information, if applicable. Data visualisation and analysis were carried out using R (v 4.1.2) [21].

**Table 1:** The criteria used in the screening for relevant literature

Screening	Inclusion criteria
First screening	Contains a tick species found in the Western Palearctic
	Contains location information in the Western Palearctic
Second screening	Publication in English
	Original, peer-reviewed work
	Tick is identified to species level
	Contains specific geographic coordinates of tick location (< 10 km accuracy)
	Tick not from a migratory or imported animal

## Results

**Fig. 1:**
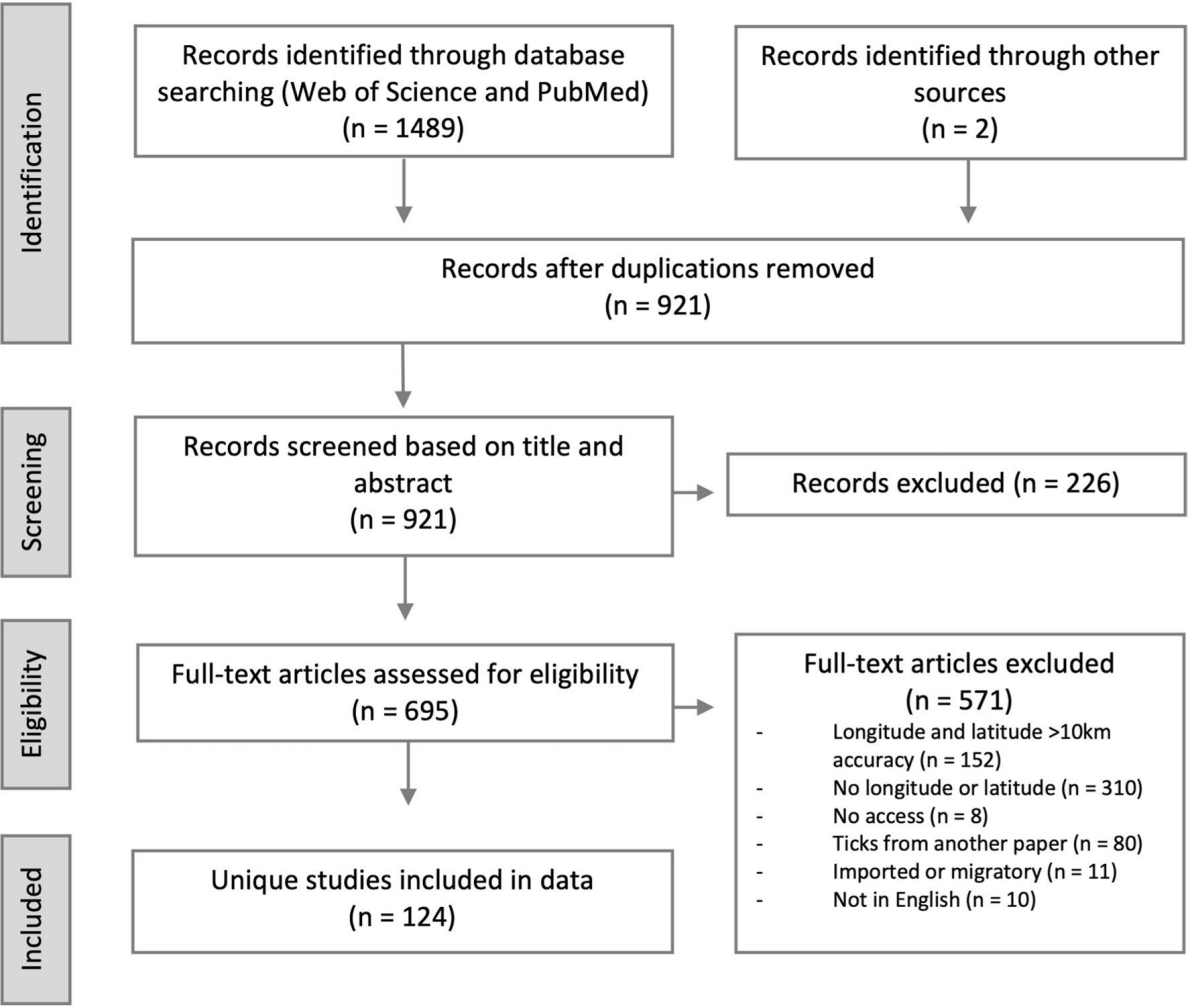
Preferred Reporting Items for Systematic Reviews and Meta-Analyses (PRISMA) flow diagram of the selection of studies for inclusion in this systematic review of geolocations of ticks in Europe between 2015 and 2021

A total of 1489 publications were identified from the literature search of the two databases: 727 from Web of Science and 762 from PubMed. Two additional publications found during the search that fulfilled the eligibility criteria were also included. Of these 1491 publications, 570 were duplicates and removed. The remaining 921 publications were screened based on their title and abstract, resulting in the exclusion of a further 226 publications. The full texts of the remaining 695 publications were then screened for eligibility; of these, 310 publications were excluded due to the failure to provide coordinates and 152 were removed due to uncertainty in the coordinates provided. In the latter case, such coordinates were for centroids of large administrative divisions, cities or national parks, or the publication referred to different sampling sites with coordinates but failed to relate this information to which species were found at each sampling site. An additional 80 publications were discarded as they did not contain original records, 11 referenced ticks from migratory animals, 10 were not available in English and eight publications could not be obtained. Following the first and second screening, a total of 124 unique studies remained for analysis that fulfilled the inclusion criteria [10, 22–144] (Fig. 1; Additional file 1: Dataset S1).

**Fig. 2:**
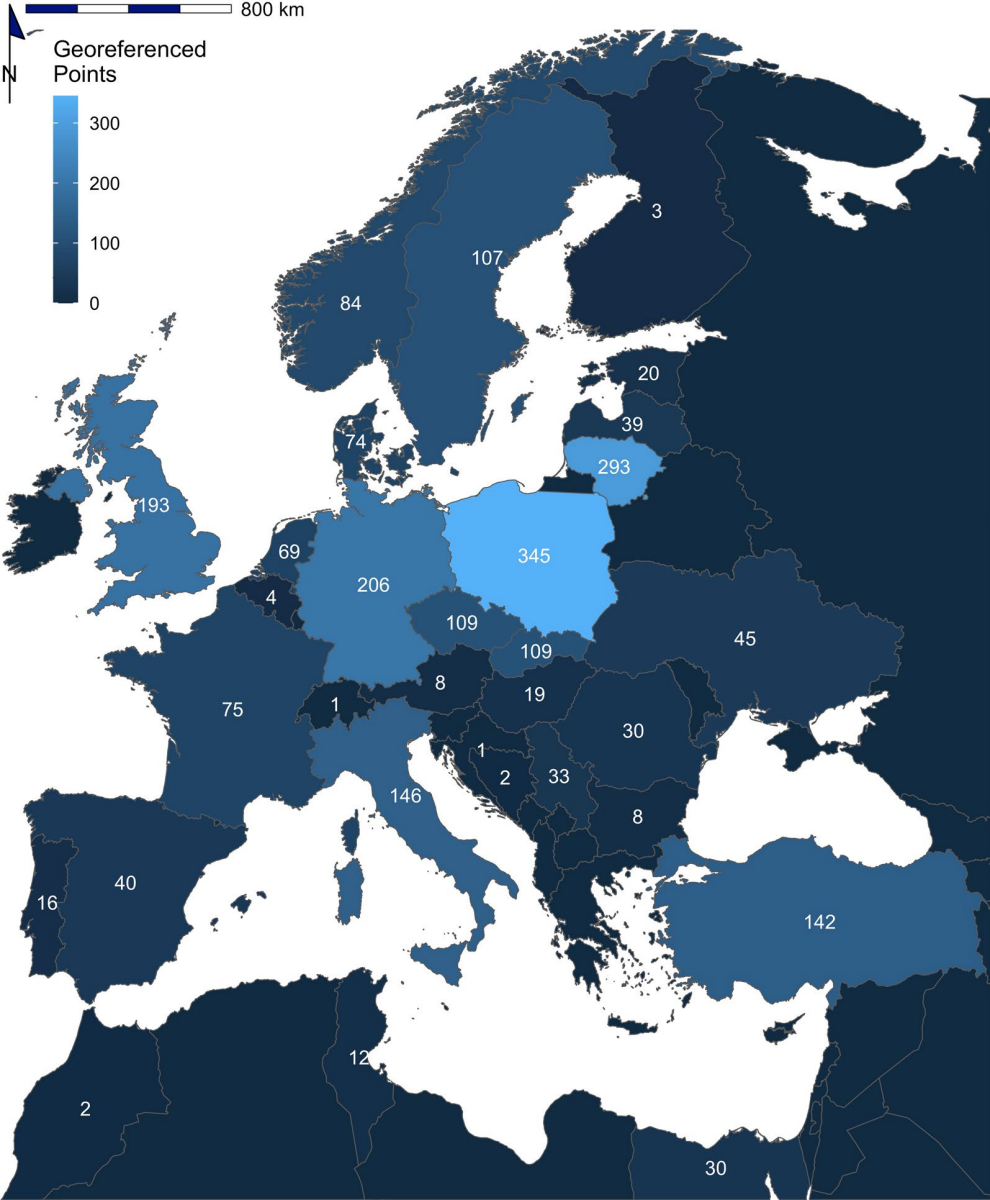
The number of georeferenced tick records found in each country in the systematic search from 2015 to 2021

**Fig. 3:**
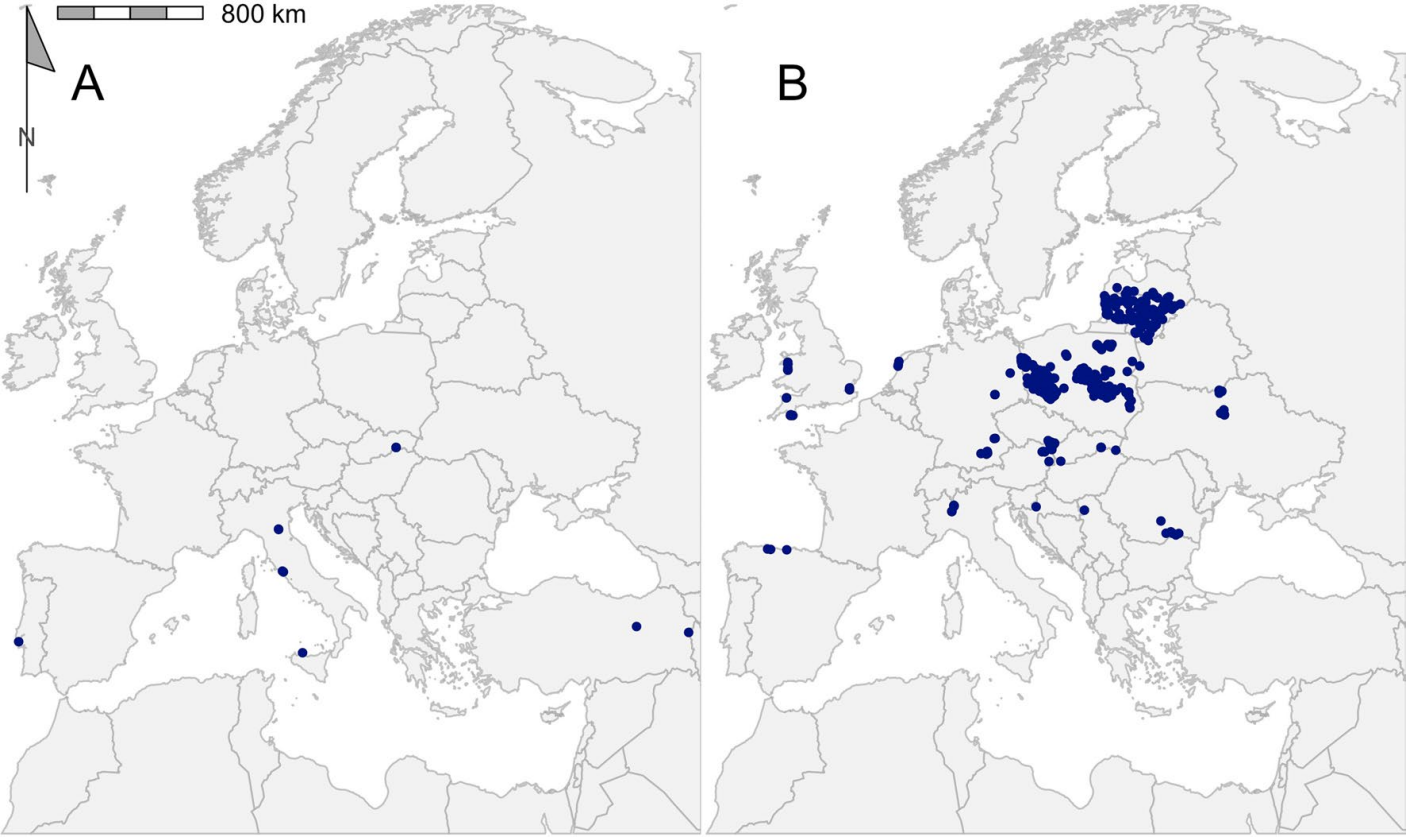
Georeferenced records of *Dermacentor* species *D. marginatus* (**a**) and *D. reticulatus* (**b**) found in the literature published between 2015 and 2021. Filled blue circles indicate recorded collected sites

**Fig. 4:**
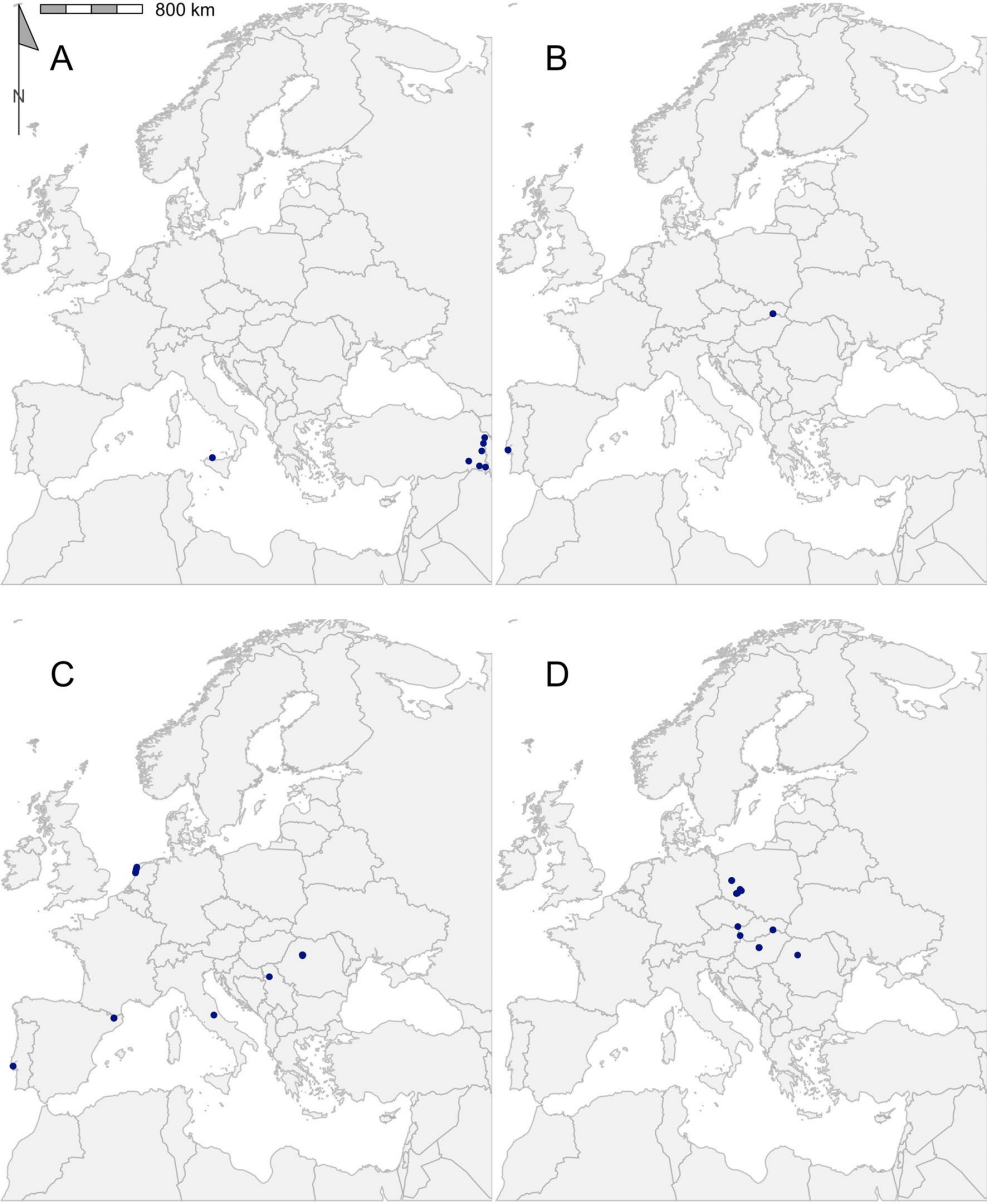
Georeferenced records of *Haemaphysalis* species *H. sulcata* (**a**), *H. inermis* (**b**), *H. punctata* (**c**) and *H. concinna* (**d**) found in the literature published between 2015 and 2021. Filled blue circles indicate recorded collected sites

**Fig. 5:**
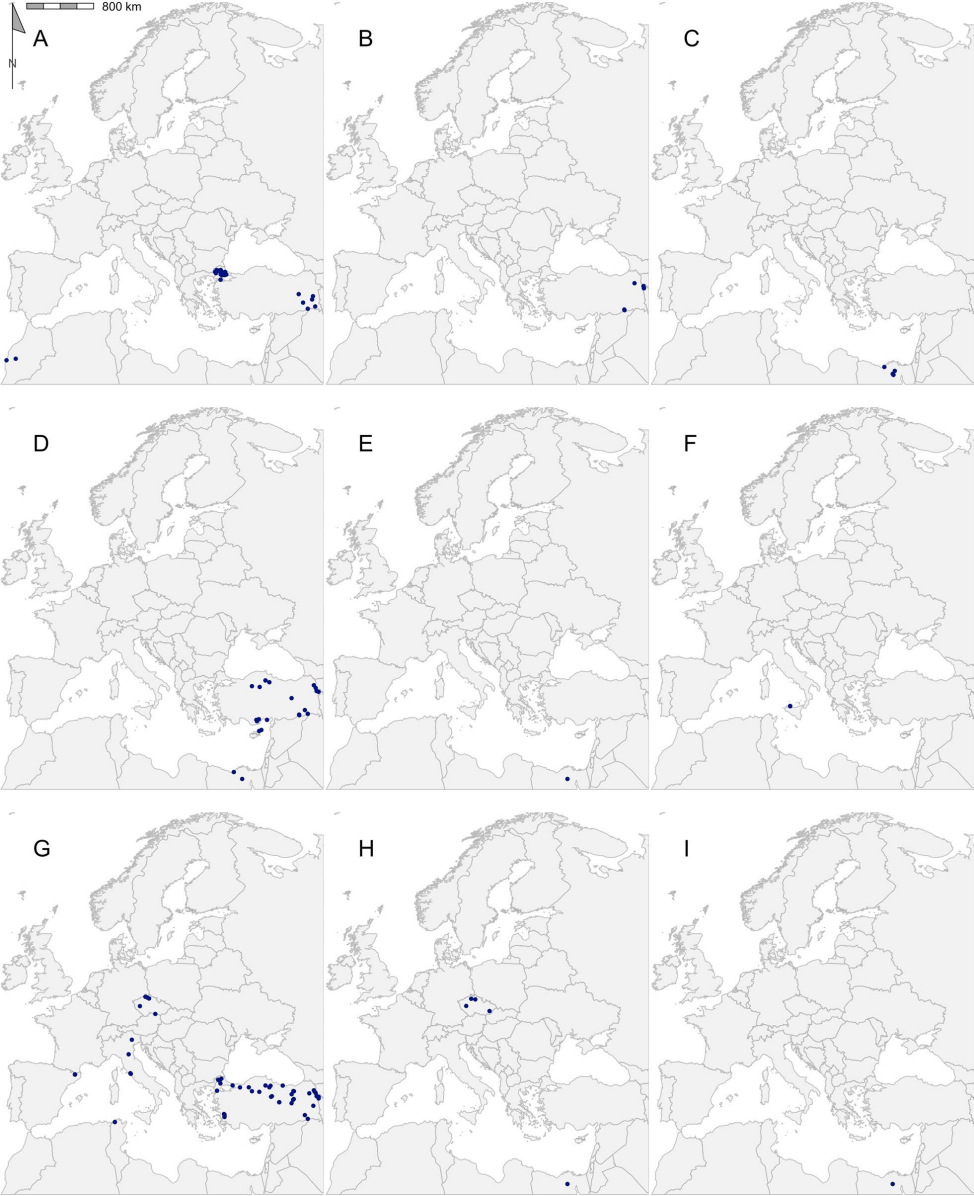
Georeferenced records of *Hyalomma* species *H. aegyptium* (**a**), *H. asiaticum* (**b**), *H. dromedarii* (**c**), *H. excavatum* (**d**)*, H. impeltatum* (**e**)*, H. lusitanicum* (**f**)*, H. marginatum* (**g**)*, H. rufipes* (**h**) and *H. truncatum* (**i**) found in the literature published between 2015 and 2021. Filled blue circles indicate recorded collected sites

**Fig. 6:**
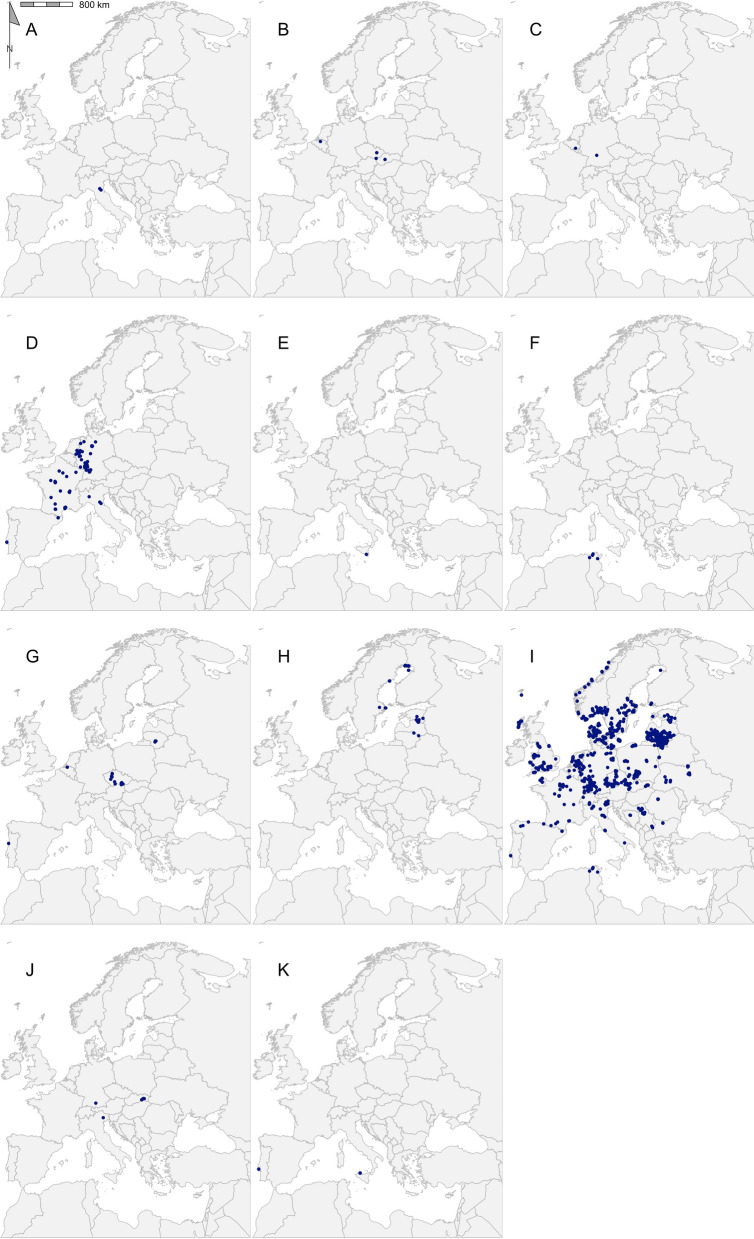
Georeferenced records of *Ixodes* species *I. acuminatus* (**a**), *I. arboricola,* (**b**), *I. ariadnae* (**c**), *I. frontalis* (**d**)*, I. gibbosus* (**e**)*, I. inopinatus* (**f**)*, I. lividus* (**g**)*, I. persulcatus* (**h**), *I. ricinus* (**i**), *I. trianguliceps* (**j**) and *I. ventalloi* (**k**) found in the literature published between 2015 and 2021. Filled blue circles indicate recorded collected sites

**Fig. 7:**
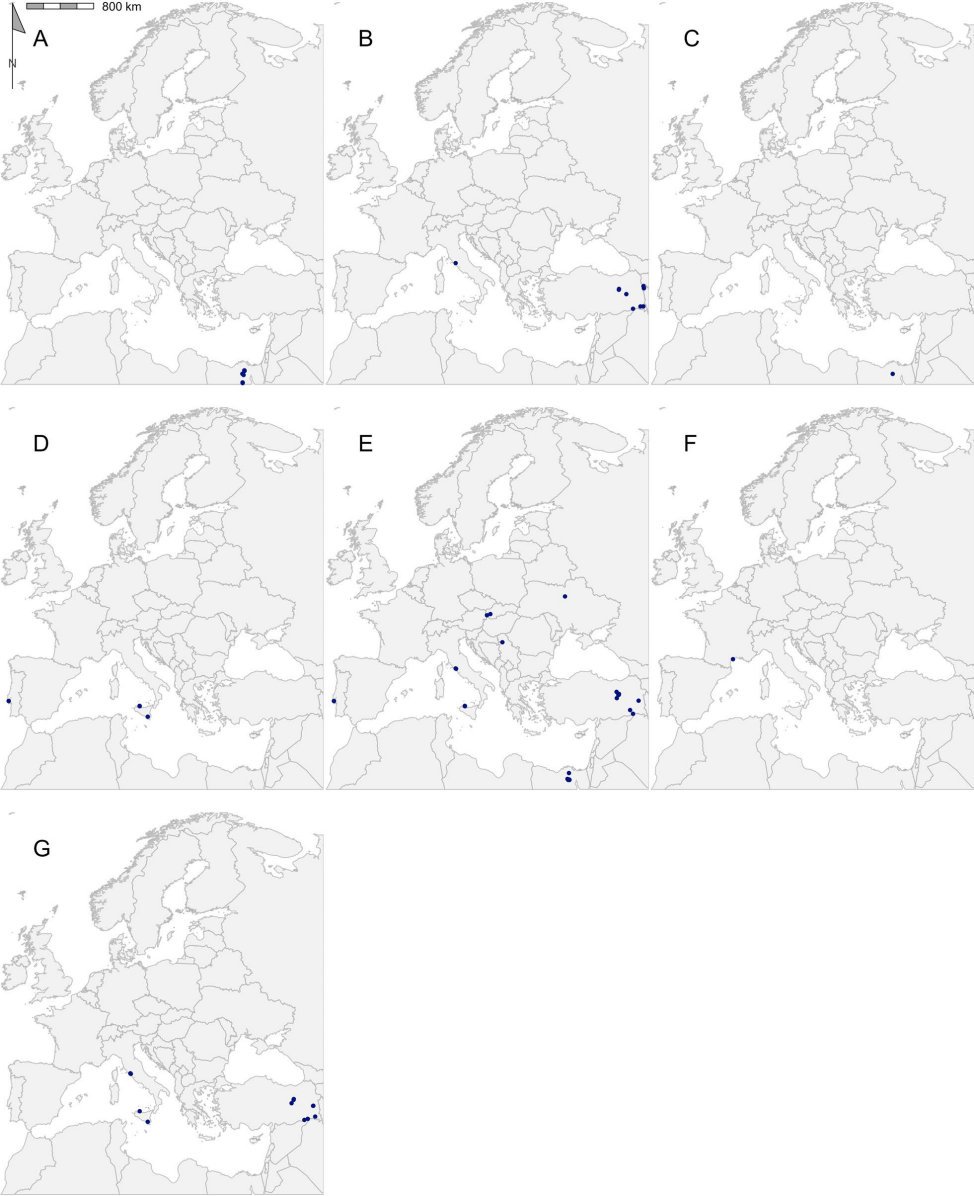
Georeferenced records of *Rhipicephalus* species *R. annulatus* (**a**), *R. bursa* (**b**), *R. pulchellus* (**c**)*, R. pusillus* (**d**)*, R. sanguineus* sensu lato (**e**)*, R. sanguineus* sensu stricto (**f**) and *R. turanicus* (**g**) found in the literature published between 2015 and 2021. Filled blue circles indicate recorded collected sites

A total of 2267 coordinate-referenced tick records from 33 tick species in 31 countries were regarded as eligible for inclusion in the final dataset (Fig. 2; Additional file 1: Dataset S1). Table 2 shows the number of records for each species, as well as the number of publications describing the location of each species. *Ixodes* was the predominant genus, accounting for 64.2% of records, followed by *Dermacentor* (22.5%), *Hyalomma* (6.6%), *Haemaphysalis* (3.5%) and *Rhipicephalus* (3.2%) (Figs. 3, 4, 5, 6, 7). In terms of individual species, the highest number of records were for *I. ricinus* (55%) (Fig. 6i), followed by *D. reticulatus* (22.1%) (Fig. 3b) and *Ixodes frontalis* (4.8%) (Fig. 6d). The number of records per species ranged from 1246 records for *I. ricinus* to just one for *Ixodes gibbosus*. All records documented as *Rhipicephalus sanguineus *sensu lato*, Rhipicephalus sanguineus* complex or simply *Rhipicephalus sanguineus* were combined and taken forward as *Rhipicephalus* *sanguineus *s.l.. *Rhipicephalus sanguineus *sensu stricto remained under this name.

**Table 2:** Geographical records of ticks found in the Western Palearctic in literature published between 2015 and 2021

Genus	Species	Records (*n*)	Publications (*n*)	Longitudinal range	Latitudinal range	Percentage of ticks from hosts	Most represented host order
*Dermacentor*	*Dermacentor marginatus*	11	7	− 09.20 to 44.02°E	38.17–48.58°N	36.36	Rodentia
	*Dermacentor reticulatus*	500	28	− 05.71 to 30.58°E	43.39–56.68°N	2.20	Rodentia
*Haemaphysalis*	*Haemaphysalis sulcata*	9	2	13.34–44.26°E	37.49–39.62°N	66.67	Squamata
	*Haemaphysalis concinna*	38	7	16.11–23.57°E	46.76–52.15°N	76.32	Passeriformes
	*Haemaphysalis inermis*	7	4	− 09.20 to 20.78°E	38.72–48.58°N	42.86	Rodentia
	*Haemaphysalis punctata*	25	6	− 09.20 to 23.59°E	38.72–53.12°N	40	Passeriformes
*Hyalomma*	*Hyalomma aegyptium*	43	4	− 09.58 to 43.57°E	31.65–41.58°N	39.53	Testudines
	*Hyalomma asiaticum*	6	2	40.79–44.16°E	37.16–40.13°N	100	Artiodactyla
	*Hyalomma dromedarii*	5	1	29.56–31.36°E	30.04–30.91°N	100	Artiodactyla
	*Hyalomma excavatum*	25	3	29.56–44.15°E	30.15–40.99°N	96	Artiodactyla
	*Hyalomma impeltatum*	1	1	31.00–31.00°E	30.15–30.15°N	100	Artiodactyla
	*Hyalomma lusitanicum*	5	1	13.34–13.35°E	38.15–38.17°N	0	–
	*Hyalomma marginatum*	59	8	02.21–44.16°E	36.97–50.75°N	89.83	Artiodactyla
	*Hyalomma rufipes*	5	2	13.57–31.00°E	30.15–50.53°N	100	Perissodactyla
	*Hyalomma truncatum*	1	1	31.00–31.00°E	30.15–30.15°N	100	Artiodactyla
*Ixodes*	*Ixodes acuminatus*	2	1	11.45–11.74°E	44.23–44.42°N	0	–
	*Ixodes arboricola*	7	2	04.52–18.87°E	48.57–51.12°N	85.71	Passeriformes
	*Ixodes ariadnae*	2	2	05.23–09.93°E	49.16–50.16°N	50	Rodentia
	*Ixodes frontalis*	108	5	− 09.20 to 11.74°E	38.72–53.01°N	37.96	Passeriformes
	*Ixodes gibbosus*	1	1	14.74–14.74°E	37.00–37.00°N	100	Lagomorpha
	*Ixodes inopinatus*	4	1	08.33–10.12°E	36.39–37.05°N	25	Artiodactyla
	*Ixodes lividus*	21	3	− 08.80 to 23.95°E	40.46–55.03°N	19.05	Passeriformes
	*Ixodes persulcatus*	42	4	17.64–27.30°E	55.78–65.78°N	0	–
	*Ixodes ricinus*	1246	89	− 09.20 to 30.58°E	36.39–66.20°N	12.12	Passeriformes
	*Ixodes trianguliceps*	11	4	10.58–21.35°E	46.04–48.76°N	72.73	Rodentia
	*Ixodes ventalloi*	8	2	− 09.20 to 13.35°E	38.15–38.73°N	0	–
*Rhipicephalus*	*Rhipicephalus annulatus*	11	1	31.03–31.42°E	29.14–30.54°N	100	Artiodactyla
	*Rhipicephalus bursa*	10	2	11.75–44.16°E	37.32–42.34°N	80	Artiodactyla
	*Rhipicephalus pulchellus*	1	1	31.00–31.00°E	30.15–30.15°N	100	Artiodactyla
	*Rhipicephalus pusillus*	9	3	− 09.20 to 14.74°E	37.00–38.73°N	11.11	Lagomorpha
	*Rhipicephalus sanguineus* sensu lato	30	8	− 09.20 to 43.25°E	30.04–50.23°N	53.33	Carnivora
	*Rhipicephalus sanguineus *sensu stricto	1	1	03.50–03.50°E	43.33–43.33°N	0	–
	*Rhipicephalus turanicus*	13	4	11.75–43.57°E	37.00–42.33°N	61.54	Artiodactyla

The full dataset is available in Additional file 1: Dataset S1

Most of the coordinate-referenced ticks were collected from vegetation (78.5%) and identified morphologically (81.2%). However, this was species dependent, with *Ixodes persulcatus* being solely found in vegetation while *Rhipicephalus* *annulatus* was exclusively collected from hosts (Table 2). Information on host order, genus or species was available for 433 records. The host order with the highest representation in this dataset was Passeriformes, followed by Artiodactyla and Rodentia.

## Discussion

This systematic review provides an updated dataset of high-resolution tick occurrence records in the Western Palearctic between 2015 and 2021 for use in spatial statistics. This dataset can be used in combination with previously collated data to investigate the recent changes in tick distribution and research [15, 16]. Although 2246 records were found, the distribution of these distribution points was restricted compared to previously reported tick distributions [145, 146]. These gaps may not reflect true absence, but are more likely the result of insufficient data due to the narrower temporal range (2015–2021), reduced records as a result of uncertain georeferencing in publications (hence excluded from this dataset) and biases in sampling effort. Only 8.3% of publications found in the systematic search provided localised coordinates for tick occurrences. This discrepancy between distributions using all tick records and distributions using only coordinate-referenced records is apparent for Portugal: according to the REVIVE study [147], *I. ricinus, Dermacentor marginatus* and *R. sanguineus* were found throughout Portugal from 2011 to 2020, but this is not reflected in this dataset (Figs. 3a, 6i, 7e). There is exceptional value in documenting the localised coordinates of tick occurrences as these can be used for spatial analysis and, consequently, there should be a drive to include this practice in all tick sampling protocols.

An additional factor to consider is that there is bias within the georeferenced records. There are obvious biases towards tick species with greater public and veterinary health implications. *Ixodes ricinus* and *D. reticulatus* received the greatest attention in terms of sampling efforts, representing 55% and 22.1% of the dataset, respectively. These species are considered among the most important in the Western Palearctic, and this importance has fuelled research into their distributions [12]. There are also spatial biases in terms of only a few countries representing the majority of records. The country with the highest representation of records was Poland (15.2% of all records), followed by Lithuania (12.9%) and Germany (9.1%). The focus on tick distribution in Poland and Lithuania may be due to the north-east expansion of ticks, especially *D. reticulatus,* into these areas, leading to increased sampling efforts for monitoring purposes [10]. Additionally, due to the selective nature of this systematic review, there will be bias towards research groups with protocols that include the documentation of site coordinates. It must be considered that the records of a species are only reflective of the areas sampled and, consequently, are not always complete in the ecological context. There needs to be a concerted effort to accurately document all tick species and to sample both endemic and novel regions.

The reliability of data is an essential factor for the use of that data in further analysis; consequently, any possible sources of error must be noted. As the majority of these ticks were morphologically identified (81%), there may be errors associated with misidentification, for example, due to the subtle morphological differences between tick species, lack of expertise of the researcher or emergence of new species. A recent study showed that 29.6% of ticks in the Western Palearctic and North Africa had been misidentified by researchers, with the genus *Rhipicephalus* having the highest misidentification rate (54%) [148]. Furthermore, the emergence of “new” species which closely resemble well-established species raises the question of uncertainty in historical records. For example, the recent description of *Ioxides inopinatus* and its similarity to *I. ricinus* means that historical reports of *I. ricinus* within the *I. inopinatus* range may be misreported [141, 149]. Reassuringly, the members of the *I. inopinatus* haplogroup recorded in this dataset were identified genetically [141].

The overall results of the literature search reported here are in agreement with previously reported data. For example, the records for *D. reticulatus* and *D. marginatus* match the overall trend described by Rubel et al. [145]; that is, *D. reticulatus* present in central and northern Europe and *D. marginatus* with a more southernly range around the Mediterranean, albeit with a narrower distributional range (Fig. 3). Of all the species reported, *I. ricinus* had the greatest range in distribution, being dispersed over 29 countries, ranging longitudinally from Lisbon, Portugal (9.2°W) to south of Kyiv, Ukraine (30.6°E), and latitudinally from Djebel Zaghouan, Tunisa (36.4°N) to Dønna, Norway (66.2°N) (Fig. 6i) [119, 124, 128, 141]. The extensive distribution of *I. ricinus* matches previous attempts to map its range [4, 15].

Due to the human and veterinary impact of ticks, it is essential that up-to-date reliable information on their distribution is recorded. It is therefore crucial that, where data privacy regulations allow, high-resolution methods, such as site-specific pairs of coordinates, are adopted by more researchers to ensure their work can be used as secondary data and hence applied to its full potential. These localised data can then be used in combination with previous tick collections to examine the changes in tick distribution in a period of rapid change, as well as provide insight into hotspots of tick research and the locations where future efforts should be focused.

## Supplementary Information

**Additional file 1: Dataset S1**. Extracted data for coordinate-referenced tick records in Europe from 2015 to 2021.

## Data Availability

Occurrence datasets are available in Supplementary Information.
